# *ARID1A* mutations protect follicular lymphoma from FAS-dependent immune surveillance by reducing RUNX3/ETS1-driven FAS-expression

**DOI:** 10.1038/s41418-025-01445-3

**Published:** 2025-01-23

**Authors:** Martina Antoniolli, Maria Solovey, Johannes Adrian Hildebrand, Tabea Freyholdt, Carolin Dorothea Strobl, Deepak Bararia, William David Keay, Louisa Adolph, Michael Heide, Verena Passerini, Lis Winter, Lucas Wange, Wolfgang Enard, Susanne Thieme, Helmut Blum, Martina Rudelius, Julia Mergner, Christina Ludwig, Sebastian Bultmann, Marc Schmidt-Supprian, Heinrich Leonhardt, Marion Subklewe, Michael von Bergwelt-Baildon, Maria Colomé-Tatché, Oliver Weigert

**Affiliations:** 1 Laboratory for Experimental Leukemia and Lymphoma Research (ELLF), LMU University Hospital, Munich, Germany; 2 Department of Medicine III, LMU University Hospital, Munich, Germany; 3 Biomedical Center (BMC), Department of Physiological Chemistry, Faculty of Medicine, LMU Munich, Planegg-Martinsried, Munich, Germany; 4 German Cancer Consortium (DKTK), Munich, Germany; and German Cancer Research Center (DKFZ), Heidelberg, Germany; 5 Laboratory for Translational Cancer Immunology, Gene Center, LMU Munich, Munich, Germany; 6 Anthropology and Human Genomics, Faculty of Biology, LMU Munich, Planegg, Germany; 7 Laboratory for Functional Genome Analysis (LAFUGA), Gene Center, LMU Munich, Munich, Germany; 8 Institute of Pathology, LMU University Hospital, Munich, Germany; 9 Bavarian Center for Biomolecular Mass Spectrometry at Klinikum Rechts der Isar (BayBioMS@MRI), Technical University Munich, Munich, Germany; 10 Bavarian Center for Biomolecular Mass Spectrometry (BayBioM), TUM School of Life Science, Technical University Munich, Munich, Germany; 11 Faculty of Biology and Center for Molecular Biosystems (BioSysM), Human Biology and BioImaging, LMU Munich, Planegg, Germany; 12 Institute of Experimental Hematology, TranslaTUM, Klinikum rechts der Isar, Technical University Munich, Munich, Germany; 13 Comprehensive Cancer Center Munich (CCCM), University Hospital, LMU Munich, Munich, Germany; 14 Bavarian Cancer Research Centre (BZKF), Munich, Germany; 15 Institute of Computational Biology, Helmholtz Zentrum Munich, German Research Center for Environmental Health, Neuherberg, Germany

**Keywords:** Cell death and immune response, Cancer microenvironment, Haematological diseases

## Abstract

The cell death receptor FAS and its ligand (FASLG) play crucial roles in the selection of B cells during the germinal center (GC) reaction. Failure to eliminate potentially harmful B cells via FAS can lead to lymphoproliferation and the development of B cell malignancies. The classic form of follicular lymphoma (FL) is a prototypic GC-derived B cell malignancy, characterized by the t(14;18)(q32;q21)IGH::*BCL2* translocation and overexpression of antiapoptotic BCL2. Additional alterations were shown to be clinically relevant, including mutations in *ARID1A*. ARID1A is part of the SWI/SNF nucleosome remodeling complex that regulates DNA accessibility (“openness”). However, the mechanism how *ARID1A* mutations contribute to FL pathogenesis remains unclear. We analyzed 151 FL biopsies of patients with advanced-stage disease at initial diagnosis and found that *ARID1A* mutations were recurrent and mainly disruptive, with an overall frequency of 18%. Additionally, we observed that *ARID1A* mutant FL showed significantly lower FAS protein expression in the FL tumor cell population. Functional experiments in BCL2-translocated lymphoma cells demonstrated that ARID1A is directly involved in the regulation of FAS, and ARID1A loss leads to decreased FAS protein and gene expression. However, ARID1A loss did not affect *FAS* promotor openness. Instead, we identified and experimentally validated a previously unknown co-transcriptional complex consisting of RUNX3 and ETS1 that regulates *FAS* expression, and ARID1A loss leads to reduced *RUNX3* promotor openness and gene expression. The reduced FAS levels induced by ARID1A loss rendered lymphoma cells resistant to both soluble and T cell membrane-anchored FASLG-induced apoptosis, and significantly diminished CAR T cell killing in functional experiments. In summary, we have identified a functionally and clinically relevant mechanism how FL cells can escape FAS-dependent immune surveillance, which may also impact the efficacy of T cell-based therapies, including CAR T cells.

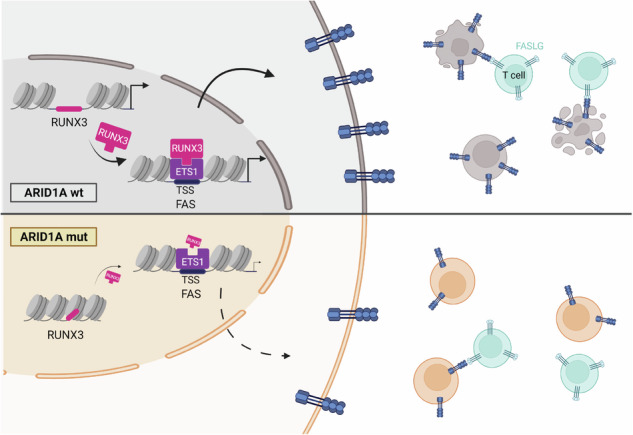

## Introduction

Avoiding immune destruction is a hallmark of cancer [1]. This is particularly prominent in malignant lymphomas, the most common type of blood cancer [2]. Most B cell Non-Hodgkin lymphomas (B-NHL) originate from germinal center (GC) B cells. GCs are highly specialized microstructures within lymphoid tissues where GC reactions occur, involving a complex interplay among B cells, T cells, and antigen-presenting cells. During the GC reaction, B cells undergo iterative rounds of genetic mutations of their immunoglobulin genes followed by selection to ultimately produce higher affinity immunoglobulins [3]. The cell death receptor FAS and its ligand (FASLG) play crucial roles in the germinal center reactions. Normal GC B cells express high levels of FAS and low levels of antiapoptotic BCL2 [4], rendering them prone to apoptosis induction. Failure to eliminate potentially harmful B cells can lead to accumulation of abnormal B cells and -eventually- the development of B-NHLs (and/or autoimmunity).

Follicular lymphoma (FL) is a prototypic GC-derived B-NHL and a clinically and molecularly highly heterogeneous disease. The molecular hallmark of classic FL is the translocation t(14;18)(q32;q21) IGH::*BCL2* [5, 6], which is acquired in early B cells [7] and leads to aberrant overexpression of BCL2. Yet, the *BCL2* translocation alone is insufficient for lymphomagenesis. Additional genetic and epigenetic alterations contribute to the development of FL and regulate critical interactions with the tumor microenvironment (TME) [8].

Many groups including us are increasingly untangling the multifaceted genetic landscape of FL [9, 10]. We have previously shown that distinct gene mutations are linked with the clinical course and treatment outcome in patients with advanced-stage FL receiving standard immunochemotherapies [11, 12], including mutations in *ARID1A*. ARID1A is part of a multimeric SWItch/Sucrose Non-Fermentable (SWI/SNF) nucleosome remodeling complex which plays a pivotal role in regulating chromatin structure [13]. By altering DNA accessibility (“openness”) it is involved in the regulation of gene expression [14–16]. *ARID1A* is recurrently mutated in FL at initial diagnosis [10, 11] with an increase from <10% in biopsies from limited-stage disease to >20% in advanced stage FL [17]. These mutations are mostly heterozygous and disruptive, leading to protein haplodeficiency [18]. However, the contribution of ARID1A loss to FL development and progression and to the biology of the disease remains unclear.

Interestingly, a previous functional genome-wide shRNA screen had shown that knock-down of *ARID1A* rescued a variety of cancer cell lines from FASLG-induced apoptosis [19]. Therefore, and because of the described interrelationships, we decided to study the link between ARID1A loss and FAS/FASLG-induced apoptosis in FL. Here, we show that *ARID1A* mutations disrupt a previously unknown regulatory network controlling FAS expression that involves RUNX3 and ETS1 and promotes a functionally and clinically relevant immune evasive phenotype in FL.

## Results

### *ARID1A* mutations are associated with reduced FAS levels in human FL biopsies

**Fig. 1:**
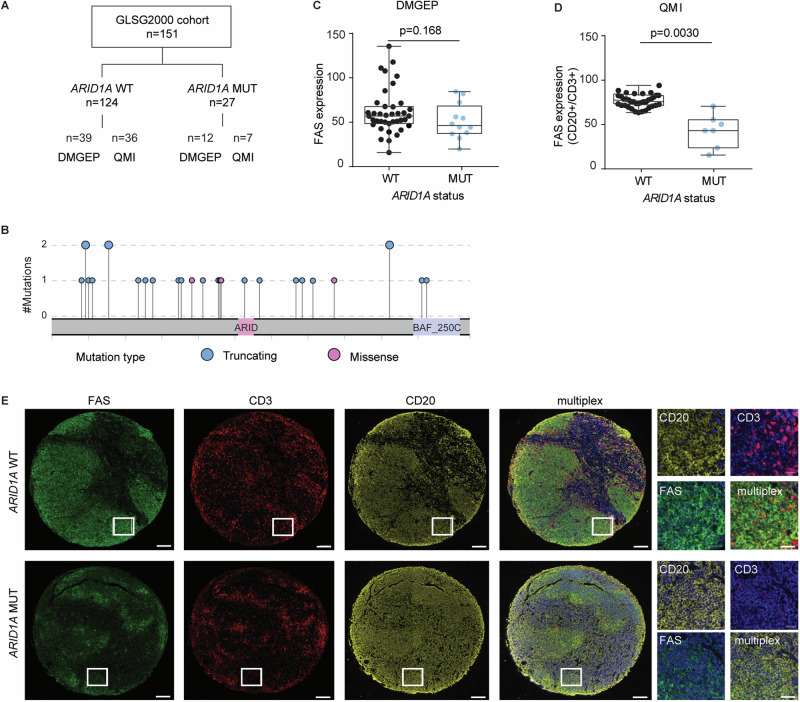
*ARID1A* mutations are associated with low FAS levels in primary human FL biopsies. **A** Schematic overview of the GLSG2000 FL cohort and available data. **B** Lollipop plot of *ARID1A* mutations in the evaluable GLSG2000 FL cohort. **C** *FAS* RNA expression in primary FL biopsies (*ARID1A*^WT^ (*N* = 39) *vs ARID1A*^MUT^ (*N* = 12)) by digital multiplex gene expression profiling (DMGEP). *P-values from Mann-Whitney U-test*. **D** FAS protein abundance in the CD20^+^ cells normalized to CD3^+^ cells in primary FL biopsies (*ARID1A*^WT^ (*N* = 36) *vs ARID1A*^MUT^ (*N* = 7)) by quantitative multispectral imaging (QMI). *P-values from Welch test*. **E** Representative multispectral images. Scale bar is 20 µm (low magnification) or 400 µm (high magnification).

First, we wanted to test the hypothesis that *ARID1A* mutations are associated with lower FAS levels in human FL. For this, we re-analyzed our previously reported cohort of diagnostic biopsies from patients with advanced stage FL at initial diagnosis [11], consisting of 151 evaluable cases with available targeted DNA sequencing data that included *ARID1A* gene mutation status. Thereof, 51 cases had also been analyzed by digital multiplex gene expression profiling (DMGEP) that included *FAS* expression levels [20], and 43 cases were available for quantitative multispectral imaging (QMI) that included staining for FAS (Fig. 1A).

The overall frequency of non-synonymous *ARID1A* mutations (at allelic fractions $\ge$ 5%) was 18% (27/151), the majority categorized as disruptive and distributed throughout the coding region of the gene (23/27, 88%) (Fig. 1B). *ARID1A* mutant (*ARID1A*^MUT^) FL (*N* = 12) showed a trend towards lower overall *FAS* gene expression levels compared to *ARID1A* wild type (*ARID1A*^WT^) cases (*N* = 39) by DMGEP (Fig. 1C). As DMGEP represents bulk gene expression data and *FAS* is known to be highly expressed by non-FL cells such as T cells and macrophages of the TME, limiting our ability to detect FAS loss in tumor cells, we performed QMI, which allows single cell resolution. Quantification of FAS protein expression in the FL tumor cell population revealed lower expression in *ARID1A*^MUT^ (*N* = 7) *vs ARID1A*^WT^ (*N* = 36) FL (Fig. 1D, E). Overall, this data shows that *ARID1A* mutations are highly recurrent, predominantly disruptive and associated with lower FAS expression in tumor cells in primary human FL.

### *ARID1A* disruption results in decreased FAS protein expression

**Fig. 2:**
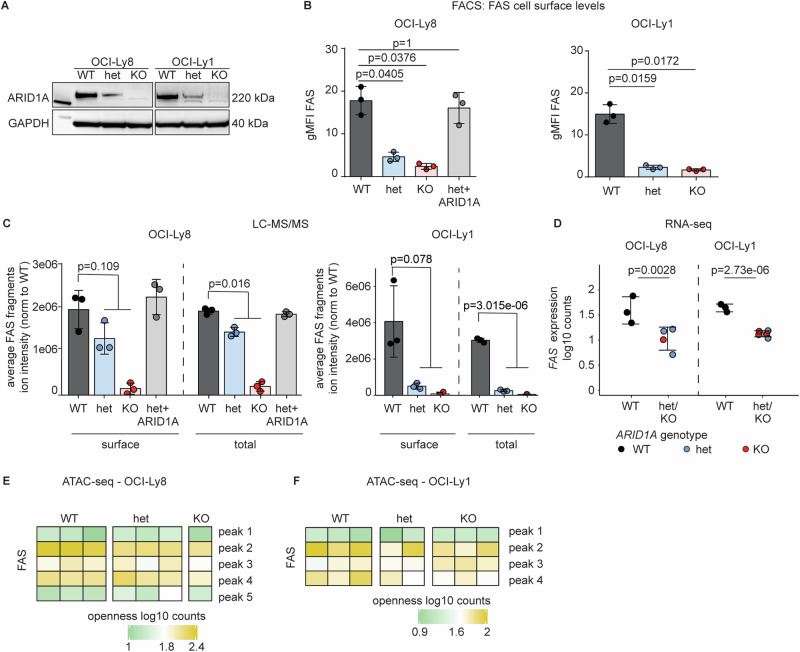
ARID1A loss results in decreased *FAS* gene expression but does not affect FAS promotor openness. **A** Western blot of single-cell derived clones of OCI-Ly8 and OCI-Ly1 cells with *ARID1A*^WT^ (WT) or CRISPR-Cas9-introduced heterozygous *ARID1A* mutation (het) or *ARID1A* knock-out (KO) (*N* = 3). **B** FAS cell surface expression on OCI-Ly8 (left panel) and OCI-Ly1 (right panel) clones by FACS. Bar diagram for the geometric means of independent biological replicates (*N* = 3). *P-values for OCI-Ly8 are from two-sided t-test, OCI-Ly1 from Welch test, Bonferroni adjusted. Each group was tested against WT*. **C** FAS peptide abundance by targeted proteomics in the surface proteome and total proteome lysates of OCI-Ly8 (left panel) and OCI-Ly1 (right panel) clones (*N* = 3). *P-values from two-sided t-test were used. Het and KO values were tested together against WT*. **D** *FAS* RNA expression by RNA-Seq in OCI-Ly8 and OCI-Ly1 single-cell-derived clones (*ARID1A*^WT^ (N = 6) and *ARID1A*^MUT^ (*N* = 9)). P-values from the DESeq2 R package for differential expression analysis with the default Benjamini-Hochberg correction were used. Het and KO values were tested together against WT. **E** FAS promotor accessibility (five detected peaks) measured by ATAC-Seq in OCI-Ly8 clones (*ARID1A*^WT^ (*N* = 3) and *ARID1A*^MUT^ (*N* = 4)). **F** FAS promotor accessibility (four detected peaks) measured by ATAC-Seq in OCI-Ly1 clones (*ARID1A*^WT^ (*N* = 3) and *ARID1A*^MUT^ (*N* = 5)). *Pooled data from biological replicates (N) are represented as mean* *±* *SD*.

For mechanistic and functional studies, we utilized human B cell lymphoma cell lines that harbor the FL-hallmark t(14;18)(q32:q21)IGH::BCL2 translocation, including two *ARID1A*^WT^ cell lines (OCI-Ly1 and OCI-Ly8) and an *ARID1A*^MUT^ cell line (Karpas422). We confirmed lower ARID1A expression in *ARID1A*^MUT^ cells by Western blot (Supplementary Fig. 1A) and flow cytometry showed corresponding lower FAS expression on the cell surface of *ARID1A*^MUT^ cell line compared to *ARID1A*^WT^ cell lines (Suppl Fig. 1B). To demonstrate that ARID1A is directly involved in the regulation of FAS expression, we introduced heterozygous (het) or homozygous (hom) *ARID1A* deletions into the *ARID1A*^WT^ cell lines OCI-Ly1 and OCI-Ly8 by CRISPR/Cas9 and generated single-cell derived clones. Immunoblotting confirmed lower ARID1A expression (*i.e*., haplodeficiency) in *ARID1A*^het^ cells, and complete knock-out (KO) in *ARID1A*^hom^ cells (Fig. 2A). Next, we evaluated FAS cell surface expression on these cells by flow cytometry. Again, we observed reduced FAS levels on *ARID1A*^het^ and KO cells (Fig. 2B). Of note, ectopic re-expression of ARID1A in *ARID1A*^het^ cells (OCI-Ly8) restored FAS levels (Fig. 2B), indicating that ARID1A is directly involved in the regulation of FAS expression. We validated lower FAS protein expression using targeted proteomics: FAS protein levels were lower in *ARID1A*^het^ and KO cells, both in cell surface proteins as well as in the total proteome fraction, and could be rescued by re-expression of ARID1A, respectively (Fig. 2C).

### ARID1A loss results in decreased *FAS* gene expression but does not affect *FAS* promotor openness

Next, we tested whether *FAS* gene expression levels were affected by ARID1A loss. Indeed, RNA sequencing showed lower *FAS* gene expression levels in *ARID1A*^het^ and KO cells (Fig. 2D).

We hypothesized that ARID1A regulates FAS protein levels by directly affecting *FAS* promotor chromatin accessibility and performed Assay for Transposase-Accessible Chromatin using Sequencing (ATAC-Seq). Differential promotor openness analysis revealed 241 and 206 differentially open (DO) peaks for OCI-Ly1 and OCI-Ly8, respectively (Table S1, 2). However, we did not detect differences in chromatin accessibility at the *FAS* promotor upon ARID1A loss (Figs. 2E, F).

### Identification of the *FAS*-regulating RUNX3/ETS1 co-transcriptional complex

**Fig. 3:**
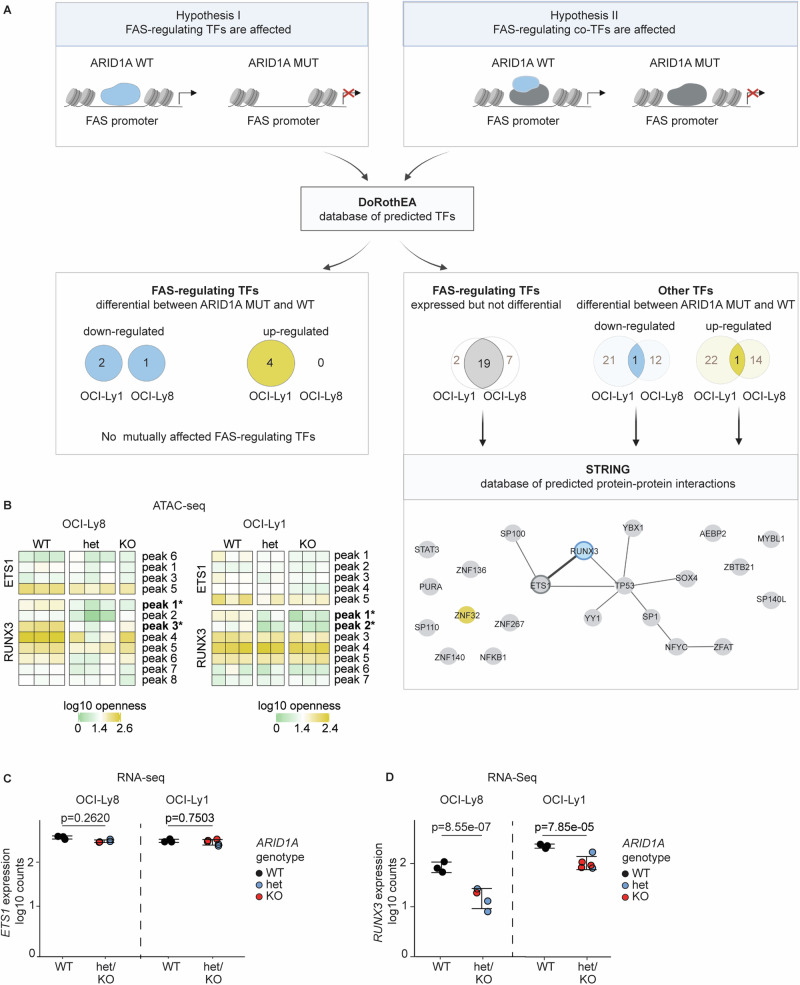
Identification of the *FAS*-regulating RUNX3/ETS1 co-transcriptional complex. **A** Analysis workflow and results of FAS-regulating transcription factors (TFs) and co-transcription factors (co-TFs). **B** ATAC-Seq differential openness analysis of *ARID1A*^MUT^ single-cell-derived clones *vs ARID1A*^WT^ control clones, in OCI-Ly8 (left panel) and OCI-Ly1 (right panel). Heatmap of log10 counts of all detected (open) peaks in the promotor regions of ETS1 and RUNX3 (*ARID1A*^WT^ (*N* = 6, in the columns) and *ARID1A*^MUT^ (*N* = 9, in the columns)), stars represent statistically significant p-values. **C** *ETS1* gene expression analysis by RNA-Seq in *ARID1A*^MUT^ clones (blue for het, red for KO) *vs ARID1A*^WT^ clones (black). **D** *RUNX3* gene expression analysis by RNA-Seq in *ARID1A*^MUT^ clones (blue for het, red for KO) *vs ARID1A*^WT^ clones (black). *P-values for* (***C***, ***D***) *were calculated using the DESeq2 package for differential expression analysis with the default Benjamini-Hochberg correction. Het and KO values were tested together against WT. Pooled data from biological replicates (N) are represented as mean* ± *SD*.

Next, we hypothesized that the lower FAS levels upon ARID1A loss may be explained by altered expression of FAS-regulating transcription factors (TFs) or co-transcription factors (co-TFs) (Fig. 3A). For this, we first used the DoRothEA database [21–23] to identify all TFs which are directly involved in the regulation of *FAS* (Table S3). We tested the differential expression of the TFs directly involved in FAS regulation in *ARID1A*^MUT^ vs *ARID1A*^WT^ cells in both OCI-Ly1 and OCI-Ly8 (Fig. 3A, “Hypothesis I”). However, we did not observe any overlap of FAS-regulating transcription factors among the down-regulated DEGs in OCI-Ly1 (MUT vs WT) and OCI-Ly8 (MUT vs WT), nor among the up-regulated DEGs between the two cell lines. (Fig. 3A). Thus, the lower FAS levels upon ARID1A loss cannot be explained by differential expression of a direct *FAS*-regulating TF. We then turned into testing the differential expression of their co-TFs (Fig. 3A, “Hypothesis II”). These co-TFs were identified as having been shown or predicted to physically interact with the *FAS*-regulating TFs, utilizing the STRING database [24] of protein-protein interactions (Fig. 3A, Table S4). In both cell lines, we identified *RUNX3* to be differentially expressed upon ARID1A loss and predicted to be an interaction partner of ETS1, which has previously been found to bind to the *FAS* promotor [25, 26]. Accordingly, the promotor openness and RNA expression of *ETS1* were unchanged (Fig. 3B, C, and Suppl Fig. 2A, C), whereas the promotor of *RUNX3* was partially closed and *RUNX3* RNA expression was reduced upon ARID1A loss (Fig. 3B, D, Table S2, and Supplementary Fig. 2B, D, E). This suggested that ARID1A loss leads to reduced *FAS* expression through reduced *RUNX3* promotor openness and reduced expression of *RUNX3*, which interacts with ETS1, a putative *FAS*-regulating TF.

### Experimental validation of the *FAS*-regulating RUNX3/ETS1 co-transcriptional complex

**Fig. 4:**
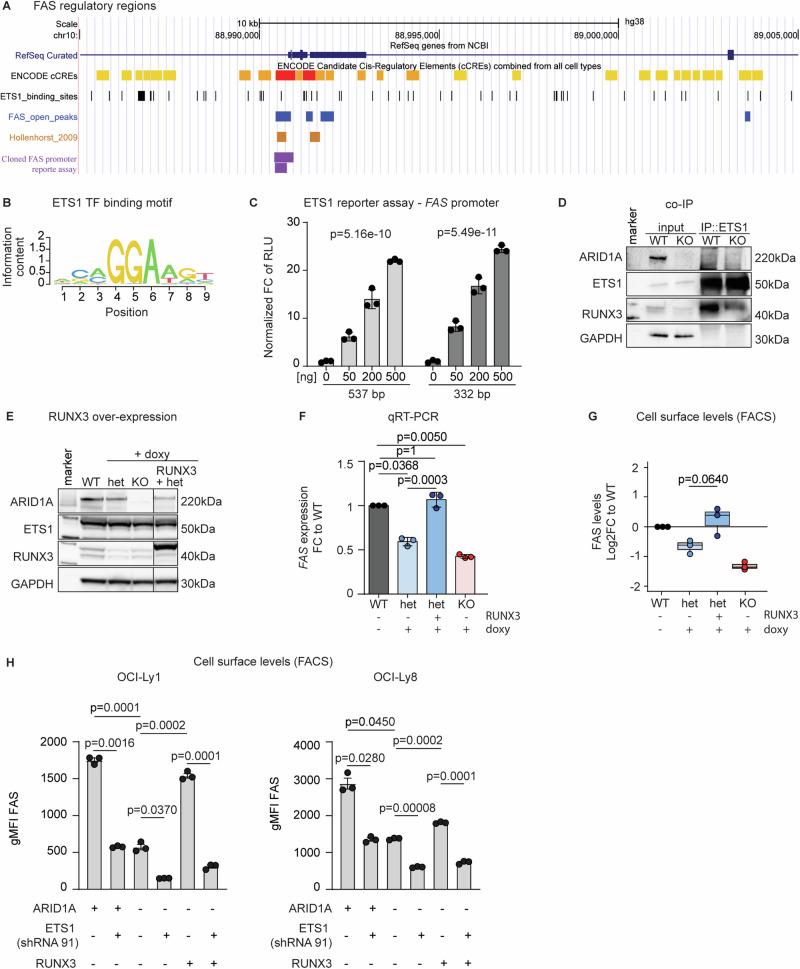
Experimental validation of the *FAS*-regulating RUNX3/ETS1 co-transcriptional complex. **A** Schematic of the *FAS* gene with annotated enhancer regions (yellow) and promotor (red), ETS1 binding sites (black), accessible chromatin regions from our ATAC-Seq data (blue), ETS1 binding sites from published ChIP-Seq data (brown) [24, 25], and the *FAS* promotor regions cloned for the reporter assay (purple). **B** ETS1 TF binding motif in *FAS* accessible promotor regions. **C** Luciferase reporter assay with co-transfection of the ETS1 expression vector and pGL3-FAS constructs in 293 T cells (*N* = 3). *P-values are from linear regression model on square root transformed dose and non-transformed response values*. **D** Western blot of inputs and ETS1-immunoprecipitated OCI-Ly1 (WT and KO) (*N* = 2). **E** Western blot of OCI-Ly8 clones (*ARID1A* WT, het, and KO) with or without stable doxycycline (dox)-induced overexpression of RUNX3 (*N* = 3). **F** Rescue of *FAS* RNA levels upon RUNX3 overexpression in OCI-Ly8 measured by quantitative real-time PCR (TaqMan assay) (*N* = 3). *P-values are from two-sided t-test, Bonferroni adjusted. All groups were tested against WT, and het* + *RUNX3 was tested against het*. **G** Rescue of FAS cell-surface protein levels upon RUNX3 over expression in OCI-Ly8 measured by FACS (*N* = 3). *P-value is from two-sided t-test. Het* + *RUNX3 was tested against het. Pooled data from biological replicates (N) are represented as mean* *±* *SD*. **H** FAS surface expression comparing *ARID1A*^WT^ (“+”), *ARID1A*^het^ (“−“) with and without ETS1-targeting shRNA (shRNA91) with and without RUNX3 overexpression in OCI-Ly1 (left bar plot) and OCI-Ly8 (right bar plot) cells by FACS (*N* = 3). *P-values are from paired Welch-test, Bonferroni adjusted*.

To functionally validate this novel FAS-regulatory network, we first analyzed the genomic context around the *FAS* promotor and searched for ETS1 binding motifs. We identified putative ETS1 binding sites that mapped to the *FAS* promotor region and overlapped with open peaks in our ATAC sequencing data as well as with previously reported ETS1 binding sites [25] (Fig. 4A, B). Then, we cloned two different sized fragments (537 bp and 332 bp) from that region into a luciferase reporter construct. The reporter constructs were expressed in HEK 293 T cells, along with increasing doses of ETS1 or RUNX3 (Supplementary Fig. 3A), which are not expressed endogenously in these cells (Supplementary Fig. 3A). With this experiment, we could show dose-dependent transactivation activity for ETS1 (Fig. 4C) confirming that ETS1 itself is a direct *FAS*-transactivating TF. In contrast, RUNX3 expression alone did not show transactivation activity (Suppl Fig. 3B).

Next, we wanted to test whether ETS1 and RUNX3 are direct interaction partners. For this, we performed co-immunoprecipitation in OCI-Ly1 cells, i.e. pull-down of ETS1 and immunoblotting for RUNX3. As shown in Fig. 4D, we could detect direct interaction of ETS1 and RUNX3. Of note, RUNX3 abundance was lower in cells with ARID1A loss (KO), both in the input and the pull-down sample (Fig. 4D). Furthermore, we confirmed that ARID1A loss (het and KO) had no impact on ETS1 protein levels in these cells, but RUNX3 levels were reduced (Fig. 4E).

Then, we overexpressed RUNX3 in lymphoma cells with ARID1A loss (het), which, unlike HEK 293 T cells, endogenously express ETS1. Immunoblotting confirmed high expression of RUNX3 while ETS1 levels were not affected (Fig. 4E, Suppl Fig. 3C). RUNX3 overexpression indeed resulted in increased *FAS* expression upon RUNX3 overexpression, both on the transcriptional level as shown by qRT-PCR (Fig. 4F, Supplementary Fig. 3D) as well as on the protein level as shown by flow cytometry (Fig. 4G, Supplementary Fig. 3E).

Finally, we aimed to directly demonstrate that ETS1 and RUNX3 cooperate in regulating FAS expression in lymphoma cells. For this, we knocked down ETS1 (ETS1 k/d) using a small-hairpin RNA (shRNA) in OCI-Ly1 and OCI-Ly8 cells and measured FAS cell surface levels by flow cytometry. ETS1 k/d effectively reduced ETS1 levels, as confirmed by Western blot (Supplementary Fig. 3F). We found that reduced ETS1 levels led to decreased FAS cell surface expression, comparable to the effect of ARID1A loss (Fig. 4H and Suppl Fig. 3G). Additionally, the ability of RUNX3 to restore FAS surface levels in *ARID1A*^het^ cells was impaired by ETS1 k/d (Fig. 4H and Supplementary Fig. 3G).

In summary, these experiments functionally validate our model of an ARID1A-dependent RUNX3/ETS1-mediated network regulating *FAS* expression.

### ARID1A loss leads to functionally relevant reduction of FAS/FASLG-induced apoptosis

**Fig. 5:**
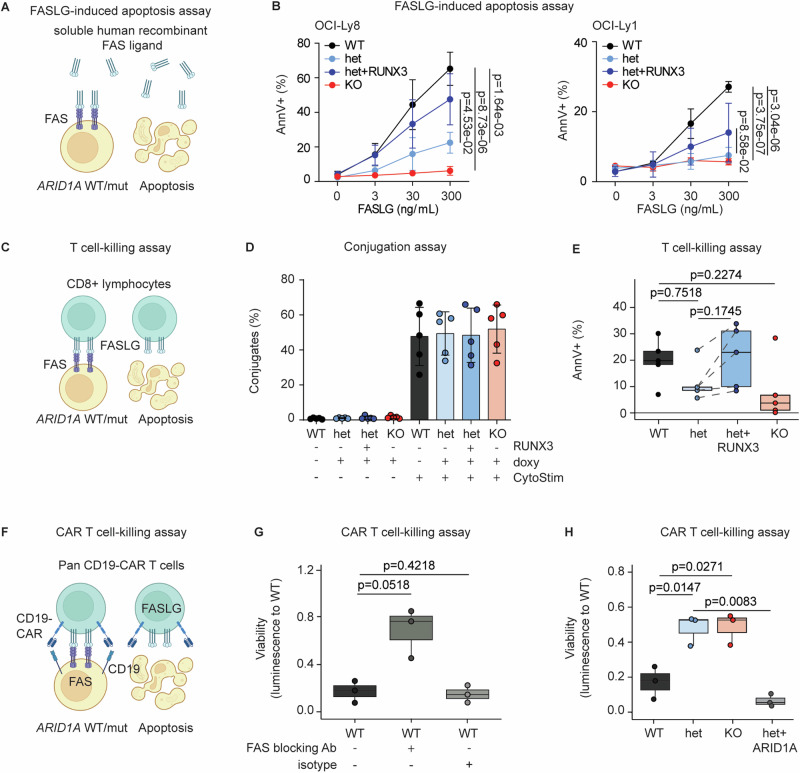
ARID1A loss leads to functionally relevant reduction of FAS/FASLG-induced apoptosis. **A** Schematic of the FASLG-induced apoptosis assay (soluble human recombinant FAS ligand treatment). **B** Percent AnnexinV-positive OCI-Ly8 (left panel) and OCI-Ly1 (right panel) clones with *ARID1A* WT, het, KO and overexpression of RUNX3 on het (het + RUNX3) after 24 h treatment with increasing dose of purified soluble human recombinant FAS ligand (*N* = 3). *P-values are from a linear regression on square root transformed values tested against WT and Bonferroni-adjusted*. **C** Schematic of the T-cell mediated killing assay. **D** Conjugate formation of OCI-Ly8 cells (CFSE^+^) and CD8^+^ T-cells (VPD^+^); double-positive conjugates (CSFE^+^/VPD^+^) quantified as percentage of total CD8^+^ T-cells (VPD^+^) (*N* = 5) at indicated conditions. **E** T cell-mediated cytotoxicity assessed by quantifying the fraction of OCI-Ly8 cells (CFSE + ) undergoing apoptosis upon co-culture with CD8+ T cells, expressed as a percentage of all measured cells under the indicated conditions (N = 5, biological replicates performed with different healthy T cell donors). *P-value is from the paired Welch test. Het* + *RUNX3 was tested against het. Pooled data from biological replicates (N) are represented as mean* *±* *SD*. **F** Schematic representation of the CAR T-cell mediated killing assay. **G** Luciferized OCI-Ly8 *ARID1A*^WT^ cells were co-cultured with CD19-CAR T cells at a 1:3 effector-to-target (E:T) ratio with or without a FAS-blocking antibody or an IgG isotype control. Luminescence was measured after 24 h and normalized to the baseline luminescence at 1 h. *P-values were calculated using the paired Welch test, comparing WT against WT* + *FAS and WT* + *EV. Pooled data from biological replicates (N* = *3; performed with CAR T-cells from different healthy donors) are represented as mean* *±* *SD*. **H** Luciferized OCI-Ly8 cells of indicated genotypes were co-cultured with CD19-CAR T cells at a 1:3 effector-to-target (E:T) ratio for 48 h. (*N* = 3, biological replicates performed with CAR T-cells from different healthy donors). *P-values are from the paired Welch test. WT was tested against het, KO and het* + *ARID1A. Pooled data from biological replicates are represented as mean* *±* *SD*.

Finally, we wanted to test whether reduced FAS expression upon ARID1A loss is functionally relevant. Upon binding of FAS ligand (FASLG), FAS oligomerizes and forms the death-inducing signaling complex (DISC), activating the extrinsic apoptotic pathway [27]. Thus, we treated cells with or without ARID1A disruption with increasing doses of purified soluble human recombinant FAS ligand and assessed apoptosis by flow cytometry (Fig. 5A). In both lines, clones with ARID1A loss were less sensitive to FASLG treatment (Fig. 5B). Of note, RUNX3 overexpression restored sensitivity towards FASLG treatment (Fig. 5B).

Normally, FAS is mostly engaged by membrane-anchored FASLG on the surface of cytotoxic cells, particularly activated T cells and NK cells [28]. Therefore, we co-cultured CFSE-labeled lymphoma cells (OCI-Ly8) with or without ARID1A loss (*ARID1A*^het^ or ARID1A^KO^ *vs ARID1A*^WT^ clones) with VDP-labeled T cells (CD8^+^) from five different healthy donors (Fig. 5C). While conjugate formation of lymphoma cells and T cells were not affected by *ARID1A* genotype (Fig. 5D), cells with ARID1A loss were less sensitive to T cell-mediated apoptosis, and overexpression of RUNX3 restored sensitivity towards T cell-mediated killing (Fig. 5E).

Finally, we investigated whether reduced FAS levels resulting from ARID1A loss impair chimeric antigen receptor (CAR) T cell killing. We co-cultured luciferized lymphoma cells, with and without ARID1A loss (het and KO), with CD19-targeting CAR T cells (Fig. 5F). Our results confirmed that a FAS-blocking antibody indeed decreased CD19-CAR T cell killing (Fig. 5G), demonstrating that tumoral FAS is functionally relevant for CAR T cell efficacy. Importantly, lymphoma cells with ARID1A loss (het and KO) exhibited significantly reduced susceptibility to CD19-CAR T cell-mediated killing, while re-expression of ARID1A in *ARID1A*^het^ cells restored CAR T cell toxicity (Fig. 5H). These data establish a direct link between ARID1A loss and diminished FAS-dependent CAR T cell efficacy.

Overall, our experiments demonstrate that ARID1A loss leads to reduced FAS levels on lymphoma cells, making them more resistant to apoptosis induced by both soluble and membrane-bound FAS ligand, including CAR T cells.

## Discussion

*ARID1A* is among the ten most commonly mutated genes in cancer [29, 30]. Its pleiotropic effects in different settings, its mutation pattern and distribution across a wide variety of different tumor entities as well as apparently conflicting functional and clinical data support the concept that ARID1A functions as a context-dependent tumor suppressor [31]. This highlights the importance of investigating its function in specific tumor types and settings.

Here, we show that ARID1A loss results in a biologically and clinically relevant immune evasive phenotype in FL by rendering tumor cells resistant to FASLG-induced apoptosis. Specifically, we identify and functionally characterize a novel *FAS*-regulating network, which involves reduced RUNX3 and ETS1-driven *FAS* expression upon ARID1A loss, which may have therapeutical implications, as discussed below.

We initially identified *ARID1A* mutations in FL more than 10 years ago, when we reported a rare case of donor-derived FLs. Following a bone marrow transplantation, both the donor and the recipient developed FLs that originated from a common precursor clone [18]. Interestingly, both FLs harbored distinct *ARID1A* mutations that each resulted in protein haplodeficiency, *i.e*. these mutations were acquired individually [18]. This convergent evolution already suggested that ARID1A loss provides a selective advantage during the development and progression of FL. Now, our functional data sheds light on the underlying biology. GC T cells express both FASLG and CD40L and the decision to kill or help largely depends on the expression of their receptors on B cells [32]. *ARID1A* mutant (pre-) malignant B cells with low FAS are more likely to evade FASLG-mediated negative selection during the GC passage, which explains the high mutation frequency in FL at initial diagnosis [10, 11]. Similarly, *ARID1A* mutant (sub-) clones may evade T cell immunosurveillance during disease progression or relapse, which would explain the additional accumulation of *ARID1A* mutations in advanced stage and relapsed/refractory (r/r) FL [17]).

In fact, accumulation of *ARID1A* mutations in r/r FL may profoundly impact the efficacy of subsequent therapies, maybe most notably of chimeric antigen receptor (CAR) T cell therapies. CAR T cells targeting CD19 have demonstrated high response rates in patients with r/r FL [33, 34]. Yet, the majority of patients will ultimately relapse and the mechanisms of treatment failure are an area of active research. There is evolving preclinical and clinical evidence that FAS loss is associated with CAR T cell failure. *E.g*., a genome-wide CRISPR/Cas9 screen identified the necessary role for FAS/FASLG in CD19-directed CAR T cell killing in an in vivo model of B cell lymphoma [35]. Importantly, this study also showed that low tumoral FAS expression was predictive of poor outcome in patients with relapsed or refractory large B cell lymphoma (LBCL) in the pivotal ZUMA-1 trial of axicabtagene ciloleucel (axi-cel) [35]. Moreover, focal deletion of 10q23.3 leading to FAS loss was found to be associated with shorter progression-free survival (PFS) and overall survival (OS) in 122 evaluable patients who received CD19-directed CAR T cells for r/r LBCL [36]. Thus, we hypothesize that other alterations that lead to clinically and functionally relevant FAS loss, including mutations in *ARID1A* as described herein and in *FAS* itself, will be enriched in patients who fail CAR T cell therapies. Indeed, while this paper was under review, a study involving 89 pediatric patients with relapsed B-NHL receiving CAR T cell therapy found that *ARID1A* mutations were associated with poorer survival compared to those without such mutations [37]. Our findings that ARID1A loss leads to reduced FAS levels can provide the mechanistic link between these observations.

While immune escape is a compelling explanation for the selection of *ARID1A* mutations in FL, the loss of ARID1A and the reduction in FAS levels may have broader effects beyond immune evasion. For example, in autoimmune lymphoproliferative syndrome type IA (ALPS-FAS), defective FAS signaling has been shown to impair mTOR activation and B cell differentiation without inducing apoptosis [38]. This suggests that FAS mediated modulation of immune processes extends beyond apoptosis and may contribute to the *ARID1A* mutant phenotype in FL. In fact, a recent study in mice demonstrated that Arid1a orchestrates B cell fate during the GC reaction, and its loss shifts GC cell fate towards immature memory B cells [39]. When combined with the BCL2 oncogene, Arid1a loss indeed promoted progression of lymphoid tumors in mice [39]. Thus, *ARID1A* mutations may have other functional consequences in addition to reducing FAS levels [39] that could be of clinical relevance in human FL and should be investigated further.

Finally, our functional data demonstrates that the FAS phenotype in cells with ARID1A loss is mediated through reduced accessibility and transcription of the *FAS*-regulating co-TF *RUNX3*. Yet, RUNX3 itself may have tumor suppressive function. For example, 45%–60% of human gastric cancers do not express RUNX3 due to hemizygous deletion and hypermethylation of the *RUNX3* promotor, and *Runx3* knock-out mice develop hyperplasia of the gastric mucosa [40]. Of note, *RUNX3* is located (in close proximity to *ARID1A*) on the distal portion of the short arm of human chromosome 1 (1p36), which is commonly deleted in FL [41, 42]. Thus, RUNX3 may be a bona fide tumor suppressor in FL and be inactivated by several mechanisms, including 1p36 deletions and/or disruptive *ARID1A* mutations. In summary, we have elucidated the molecular mechanism of reduced FAS expression in *ARID1A* mutant lymphoma cells and demonstrated that this promotes functionally and potentially clinically relevant escape from T cell mediated killing.

## Materials and methods

### Primary patient samples

Gene mutation data and gene expression data from diagnostic biopsies were derived from our previously reported cohorts of patients with previously untreated advanced stage FL [11, 20]. All studies on human material were covered by IRB approvals (LMU #223-14, LMU #276-14, LMU #056/00, and LMU #539-15 fed).

### Multispectral imaging analysis (Vectra® Polaris System)

Multiplex immunohistochemistry was performed as described previously [20]. We used the following antibodies: FAS-R (EP208; AC-0178RUO; Abcam, Cambridge, UK; 1:100), CD3 (SP7; RBK024-05; Zytomed System, Berlin, Germany; 1:150); CD20 (L26; 120M-85; Cell Marque, Rocklin, CA, USA; 1:200). Images were acquired using the quantitative slide scanner with the Vectra® Polaris 1.0. (PerkinElmer, Waltham, MA, USA) and analyzed using InForm 2.4.2 (PerkinElmer) and HALO® software (Indica Labs, Albuquerque, NM, USA). Antibody details are listed in Table S6.

### Cell lines

Karpas422 cells carry a heterozygous *ARID1A* mutation [43] (Q1959fs; confirmed by Sanger sequencing) and were cultured in RPMI 1640 (PAN^TM^ Biotech, Aidenbach, Germany). OCI-Ly1 and OCI-Ly8 are *ARID1A* wild type (confirmed by targeted deep sequencing of all exons) and were cultured in IMDM (PAN^TM^ Biotech), each supplemented with 10% heat-inactivated FBS (PAN^TM^ Biotech). All cell lines were authenticated by STR profiling (Eurofins, Val Fleuri, Luxembourg) and were tested negative for mycoplasma (MycoAlert PLUS mycoplasma detection kit, Lonza, Basel, Switzerland).

### CRISPR/Cas9-mediated knock down of ARID1A

Single guide RNA (sgRNA) targeting *ARID1A* (Table S5) were cloned into the pSpCas9(BB)-2A-GFP backbone (PX458, Addgene plasmid #48138) as previously described [44]. OCI-Ly8 or OCI-Ly1 cells were transduced with Nucleofector^TM^ Solution V (Lonza) and the Nucleofector^TM^2b (Lonza) and single-cell sorted for GFP after 48 h. To validate the *ARID1A* genotype, we amplified a 439 bp fragment by PCR (30 cycles; annealing temperature 52 °C) using specific primers (Forward: GTTGAAATGCCTGTGTGGCA; Reverse: CAATATGCCACCTCAGGTTGG). The PCR products were ligated into a pCR2.1-TOPO vector (Invitrogen; Ca.No: K202020) and transformed into competent *E. coli* (Invitrogen, Ca.No: C404003). Up to 10 single colonies were Sanger sequenced. Alternatively, we amplified a 336 bp fragment around the sgRNA-targeting site (Forward: GCAGCAAAGCCAGCCTTGCTCT; Reverse: GCCATAAGGTGGGATGCCAGGC) using iProof™ High-Fidelity DNA Polymerase and confirmed *ARID1A* alterations by Sanger sequencing (Eurofins Genomics). If Sanger sequencing results were inconclusive, we submitted purified PCR products for amplicon-EZ deep sequencing with Genewiz® (Azenta Life Sciences). *ARID1A* wild type (WT) and knock-out (KO) status was defined by the presence of only wild type or mutant reads, respectively, at the sgRNA-targeted intron-exon boundary upstream of exon 8. Heterozygous (het) status showed both wild type and mutant reads. *ARID1A*^het^ and KO clones were confirmed by reduced or absent ARID1A expression compared to WT clones via Western blot.

### Construction of lentiviral vectors and cell lines

Overexpression of ARID1A was performed by lentiviral transduction (pHAGE-CMV-MCS-ARID1Awt-IHRES-ZsGREEN) as previously described [20]. To express *RUNX3* we used the lentiviral construct pTet-O-RUNX3-T2A-PuroR (Addgene # 162349). To knock down ETS1 in OCI-Ly1 and OCI-Ly8 cells, we overexpressed two commercially available ETS1 CDS-targeting shRNAs (TRCN0000005591, TRCN0000005592) using the pLKO.1-CMV-Neo vector. Cells were selected by adding 1 mg/mL geneticin (G418, Invivogen) to the medium for 14 days. A GFP-targeting shRNA (Addgene #72571) was overexpressed as a control.

### Flow Cytometry

Cells washed with PBS (PAN) and stained with anti-CD95 (FAS) antibody for 45 min at 4 °C (PE anti-human CD95 (FAS) mouse DX2 (# 305608) BioLegend (San Diego, CA, USA) (1:25). Cells were washed and resuspended in 200 µL PBS for FACS analysis. At least 10,000 events were recorded. Antibody details are listed in Table S6.

### Quantitative real-time PCR analysis

Total RNA was isolated using Direct-zol^TM^ RNA Kits (Zymo Research, Irvine, CA, USA) and transcribed into cDNA using iScript cDNA Synthesis Kit (BioRad, Hercules, CA, USA). PCR reactions were performed using TaqMan^TM^ Fast Advanced Master Mix, FAS (Hs00236330_m1 FAM-MGB FAS), RUNX3 (Hs01091094_m1 FAM-MGB RUNX3), and TBP (Hs00427620_m1 VIC-MGB TBP) assays (Thermo Fisher Scientific). The statistical analysis was done on RT-qPCR cycle threshold (ct) values normalized to housekeeping gene.

### Western Blot

Cells were lysed with radio immunoprecipitation assay buffer (RIPA). Protein concentrations were quantitated with Pierce BCA assay (Thermo Fisher Scientific). Proteins were separated on 4–12% SDS-PAGE gels. Primary antibody incubation was performed overnight at 4 °C, followed by a secondary horseradish peroxidase-conjugated antibody at room temperature for 1 h. The following antibodies were used: ARID1A, rabbit (#HPA005456), Sigma Aldrich (St. Louis, MO, USA) (1:2500); RUNX3, mouse, clone R3-5GA (#697901), BioLegend (1:2000); ETS1, rabbit, clone D8O8A (#14069S), Cell Signaling (1:2000); GAPDH, mouse, clone 6C5 (#MA5-15738-D680), Thermo Fisher Scientific (1:10000). Antibody details are listed in Table S6.

Uncropped Western blots are provided in Supplemental Material.

### Co-immunoprecipitation (IP)

Cells were lysed using Pierce^TM^ IP Lysis Buffer (Thermo Fisher Scientific) supplemented with protease and phosphatase inhibitor cocktails (Roche, Basel, Switzerland). IP was performed with 3 mg of protein. SureBeads^TM^ Protein A Magnetic Beads (BioRad) were coupled with anti-ETS1 antibody (CS#14069, Cell Signaling, Danvers, MA, USA) for 3 h at 4 °C with continuous rotation. Lysates were incubated with the bead-bound antibody overnight at 4 °C with continuous rotation. Bead-bound immunoprecipitates were washed three times with IP buffer and eluted twice with a total volume of 2x Laemmli Buffer (BioRad). Input samples (30 µg) and co-IP samples were subjects to western blot analysis. Antibodies are listed in Table S6.

Uncropped Western blots are provided in Supplementary Material.

### Mass Spectrometry

Cell surface proteins were isolated by using Pierce Cell Surface Protein Isolation Kit (Thermo Fisher Scientific, cat#89881), following the manufacturers’ instructions. The bound proteins were eluted with 350 µl Novex^TM^ Tris-Glycine Native Sample Buffer (2X) (Thermo Fisher Scientific), supplemented with DTT to a final concentration of 50 mM. In-gel trypsin digestion was performed according to standard procedures [45].

For total proteome analysis, cells were lysed in 2% SDS and 50 mM Tris-HCl pH 7.5 and heated to 95 °C for 5 min. 1 μL 100 TFA was added to each sample to hydrolyze DNA and the pH subsequently adjusted to 8.5 with 3 M Tris solution. SP3 cleanup was performed according to the protocol by Hughes et al. [46] followed by tryptic digestion overnight and stage-tip desalting. Dry peptides were reconstituted in 2% (v/v) acetonitrile, 0.1% (v/v) formic acid in HPLC grade water and spiked with PROCAL retention time standard peptide mix [47].

Liquid chromatography-coupled mass spectrometry (LC-MS/MS) analysis was performed on a Q Exactive HF-X Orbitrap (Thermo Fisher Scientific) coupled on-line to a Dionex Ultimate 3000 RSLCnano system (Thermo Fisher Scientific). FAS protein abundance was monitored using a parallel reaction monitoring assay (PRM) with a 50 min linear gradient. The recorded RAW files were imported into Skyline (64-bit, v.20.2.0.343) for data filtering and analysis. For FAS protein detection the six most intense and unique FAS peptides were selected. A spectral library was constructed using the PROSIT prediction algorithm implemented in Skyline with standard settings [48, 49]. Peaks were integrated using the automatic settings followed by manual curation of all peak boundaries. Peaks with a dotp product < 0.7 compared to the predicted peptide spectrum were excluded from analysis. For FAS protein quantification the area of all fragment ion traces over all peptides was summed.

### FAS ligand (FASLG)-induced apoptosis assay

*ARID1A* wild-type and mutant cell lines were treated with 0, 3, 30, or 300 ng/mL purified soluble human recombinant FAS ligand (SUPERFASLIGAND®, Enzo Life Sciences, Inc., Farmingdale, New York, USA) for 24 h. The cells were assayed by flow cytometry (BD FACSCanto™ II). We utilized the AnnexinV Apoptosis Detection Kit I (BD PharmingenTM, San Diego, CA, USA) and DAPI (BD PharmingenTM). The data was analyzed with the FlowJo v10 software (BD PharmingenTM).

### Co-culture assays

Lymphoma cells were stained with 1 μM CellTrace^TM^ CFSE Cell Proliferation Dye (Thermo Fisher Scientific, Waltham, Massachusetts, USA) for 4 min at room temperature (RT). CD8^+^ T cells were isolated from human peripheral blood using EasySep^TM^ Human CD8^+^ T Cell Isolation Kit (Stemcell Technologies, Vancouver, BC, Canada) and stained with 10 μM CellTrace^TM^ Violet Cell Proliferation Kit (VPD) (Thermo Fisher Scientific) for 10 min at 37 °C. CytoStim^TM^ (Miltenyi Biotec, Cologne, Germany) was used according to the manufacturers’ recommendations to activate T cells by binding the T cell receptor (TCR) and crosslinking it to major histocompatibility complex (MHC) molecules of B cells. Cells were cultured in TexMACS media (Miltenyi Biotech) in a 1:1 ratio at 37 °C up to 3 h, stained with AnnexinV and assayed by FACS as described above. Conjugate formation was calculated as [CFSE^+^/VPD^+^ cells $\div$ VPD^+^ cells] $\times$ 100%. The specific CD8^+^ T cell-mediated cytotoxity (T cell-killing assay) was calculated by subtracting the unspecific AnnexinV^+^ lymphoma cells (*i.e*., cells cultured without T cells as a control) from AnnexinV^+^ CFSE-labeled lymphoma cells co-cultured with CD8^+^ T cells, and quantified as a percentage of all measured cells as previously described [50].

### CAR-T cell co-culture assay

Single-cell-derived clones of OCI-Ly8 cells (*ARID1A* WT, het, KO, and het with ARID1A overexpression (het+ARID1A)) were lentivirally transduced with m-Cherry-tagged eFly luciferase and FACS-sorted for m-Cherry. Second-generation CD19-CAR T cells were provided by M.Sub. and generated using a pMP71 backbone with a CD28 co-stimulatory domain [51]. OCI-Ly8 cells were co-cultured with CD19-CAR T cells in TexMACS (Miltenyi Biotech) at effector-to-target (E:T) ratio of 1:3 in 96-well U-bottom plates, with 100,000 cells in 200 µL/well, at 37 °C for up to 48 h in the presence of CytoStim™ (Miltenyi Biotec). An anti-FAS neutralizing antibody (clone ZB4, Cat: 05-338, Lot: #4057957, Merck) was used at 250 ng/mL, and a mouse IgG1 Kappa isotype control (Clone: MOPC-21, Cat: 557872, BD Pharmingen™) served as control. Bright-Glo™ Luciferase reagent (Cat: E2610, Promega) was added after 1, 24, and 48 h, and luminescence was analyzed on a GloMax® Discover Multimode Microplate Reader (Promega). Killing was assessed by normalizing luminescence to the 1-h time point.

### Luciferase Reporter Assay

Two regions of *FAS* promotor (537 bp and 332 bp) were cloned into pGL3-basic vector following the manufacturer’s instruction (#E1751, Promega, Walldorf, Germany). The cloned regions (FASprom_P1 and FASprom_P2, Table S5) contained ETS1 TF binding motifs [25]. The cells were analyzed by Dual-Glo® Luciferase Assay (Promega #E2920).

### RNA-Sequencing

RNA-sequencing was performed on 8 or 7 different clones for OCI-Ly1 and OCI-Ly8, respectively, each clone being sequenced in two technical replicates. RNA was isolated using Direct-zol^TM^ RNA MicroPrep kit (Zymo Research) and a total of 10 ng of RNA per sample were subjected to RNA sequencing using a version of the prime-seq protocol [52] Illumina paired end sequencing was performed on an HiSeq 1500 (Illumina, San Diego, CA, USA) instrument. The first 16 bp read was used for sample barcode and UMI, the second 50 bp read was used for the gene. Raw data was demultiplexed using deML [53] and processed using the zUMIs pipeline [54] with STAR [55]. Reads were mapped to the human genome (hg38) with Ensemble gene annotations (GRCh38.84) and annotated using biomaRt (v2.44.4). Genes with the mean raw counts less or equal to 5 counts were filtered. Technical replicates were collapsed using the collapseReplicates() function of the DESeq2 package (v1.28.1). Counts were normalized using DESeq2 package (v1.28.1). Differentially expressed genes were calculated with DESeq2 package (v1.28.1) between the controls and mutants (het + KO). Only genes that had log2 fold change higher than 1 or lower than -1, as well as the adjusted p-value lower than 0.1 were considered as significantly differentially expressed (Table S7).

A total of fifteen single-cell-derived clones were assayed in OCI-Ly1 and OCI-Ly8. Control clones (3 per cell line) were transduced with the empty pSpCas9(BB)-2A-GFP backbone. In OCI-Ly1, two heterozygous and three ARID1A knock-out clones were sequenced. In OCI-Ly8, three heterozygous and one knock-out clone were sequenced.

### ATAC-Sequencing

Transposased fragments were prepared and pre-amplified as described in the optimized Omni-ATAC protocol [56]. Transposased DNA was amplified using Nextera XT Index pair (i5 Index Name, Illumina), specific for every sample (list in Table S8), and the KAPA HiFi PCR Kit (Roche Diagnostics), as recommended by the manufacturer. Amplified fragments were purified and eluted. Illumina paired end sequencing was performed on an HiSeq 1500 instrument, where the first 16 bp read covered the sample barcode and UMI, and the second 50 bp read was used to identify the gene. Reads were trimmed using TrimGalore (v0.6.5; default parameters), aligned to the reference genome (hg38) with Bowtie2 (v2.3.5; --very-sensitive -X 2000) and sorted by position and indexed using samtools (v1.2). Those mapping to mitochondria, mapping to less than 6 bases or with a MAPQ below 10 were removed. Genome version for alignment was GRCh38/hg38. Alignments of fragments longer than 150 bp were also filtered using Deeptools-alignmentSieve (v3.3.1). Peak calling was done using MACS2-callpeak (v2.2.6; --nomodel --keep-dup 1 -g mm), and peaks overlapping with the blacklisted ones (https://sites.google.com/site/anshulkundaje/projects/blacklists#TOC-Downloads; https://www.encodeproject.org/annotations/ENCSR636HFF/) were filtered using bedtools-intersect (v2.29.2; -v). The remaining peaks were sorted (bedtools sort) and merged (bedtools merge) into non-overlapping peaks. A consensus peak set was generated by first finding a consensus peakset per condition, and then a global consensus peakset for all conditions using the last ones with bedtools sort and merge. The final counts of aligned reads was done using featureCounts (subread package, v1.6.4).

Only peaks with more than 50 counts in at least one sample were kept. Counts were normalized using the DESeq2 package (v1.28.1). Peaks were annotated using the the ChIPseeker package (v1.24.0). Differentially open peaks were calculated between the controls and mutants (het^+^KO) using the DESeq2 package (v1.28.1). Only peaks that had log2 fold change higher than 1 or lower than -1, and an adjusted p-value lower than 0.1, were considered as significantly differentially open (Table S1).

### FAS-regulating transcription factor (TF) and co-transcription factor (co-TF) analysis

FAS-regulating TFs were retrieved from the DoRothEA database (v1.0.1) [21–23] using the expressed genes per cell line, yielding 29 and 30 FAS-regulating TFs in OCI-Ly1 and OCI-Ly8 respectively (Table S3), with 21 FAS-regulating TFs expressed in both cell lines. No mutual differentially expressed FAS-regulating TF for OCI-Ly1 and OCI-Ly8 were found among the down-regulated OCI-Ly1 DEG (MUT vs WT) and the down-regulated OCI-Ly8 DEG (MUT vs WT). Same is true for up-regulated OCI-Ly1 DEG and OCI-Ly8 DEG.

Only the 21 FAS-regulating TFs which were present in both cell lines were selected for further analysis. Next, a list of all other transcription factors that were expressed in the RNA-seq data was constructed using the DoRothEA database (v1.0.1) [21–23]. Only the TFs which were mutually differentially expressed in both OCI-Ly1 and OCI-Ly8 were considered for further analysis, which left 2 down-regulated and 1 up-regulated TFs (Table S4). Lastly, the STRING database (v11-0b) [24] was used to construct a network of protein-protein interactions (physical interactions only) between the three mutually differential TFs and the 21 FAS-regulating TFs. Only 8 out of these 21 proteins were predicted to interact with the down-regulated (but not the up-regulated) TFs.

### ETS1 binding motif identification

ETS1 binding motif sequence was identified using MotifDB (v.1.40.0) and seqLogo (v.1.64.0) R packages with Hocomoco database [57] (Fig. 4B). The coordinates of the ETS1 binding motifs in the FAS promoter region were identified using JASPAR2020 (v.0.99.10) R package (see “ETS1_binding_to_FAS_promoter” on https://github.com/colomemaria/ARID1A_follicular_lymphoma).

### Statistics

All experiments were done in replicates as indicated. Replicate data is displayed with individual data points, mean and standard deviation (SD). For co-culture experiments, T cells from different donors were used as biological replicates. Data visualization was performed with GraphPad Prism version 6.07 for Windows (GraphPad Software, San Diego, California USA) or in R (v4.2.2) ggplot2 package (v3.4.2). Statistical analysis was performed with R (v4.2.2). The choice of the test, as indicated in the corresponding figure legends, was based on whether the assumptions of the normality of the distributions and homogeneity of the variance between groups were met. These assumptions were tested with the shapiro.test() R function bartlett.test() R function for normally distributed data or flinger.test() R function for non-normally distributed data, respectively. For normally distributed data with homogeneous or non-homogeneous variance, the two-sided t-test (t.test() R function) or the Welch test (t.test() R function with the ´var.equal´ argument set to FALSE) was used, respectively. For non-normally distributed data with homogeneous variance, the Mann–Whitney U-test (wilcox_test() R function from coin package (v1.4-3)) was used. In case of multiple testing, Bonferroni correction was applied, as indicated in the figure legends. Analysis of RNA and ATAC-sequencing was done with R (v4.2.2) DESeq2 package (v1.38.1) and is described in detail in the respective sections above. For luciferase reporter assay (Fig. 4B), as well as dose-increasing FASLG-induced apoptosis (Fig. 5B) testing, linear regression on square root transformed values with lm() R function, with Bonferroni adjustment if needed, was used.

### Cartoon representations

All cartoon representations were created with BioRender.com.

## Supplementary information

Supplementary File

Supplemental Table 1

Supplemental Table 2

Supplemental Table 3

Supplemental Table 4

Supplemental Table 5

Supplemental Table 6

Supplemental Table 7

Supplemental Table 8

Supplemental Figure WB full lenght

## Data Availability

The RNA-sequencing raw data and gene expression matrices have been deposited in the Gene Expression Omnibus (https://www.ncbi.nlm.nih.gov/geo/) under accession number GSE230036. The ATAC-sequencing raw data and peak matrices have been deposited in the Sequence Read Archive (SRA) (https://www.ncbi.nlm.nih.gov/sra) under accession number PRJNA966144. The Proteomic data has been uploaded to ProteomeXchange (https://www.proteomexchange.org) under the identifier PXD041408 and can be accessed on the Panorama web repository server https://panoramaweb.org/2mQCoX.urls. The analysis code is available on Github https://github.com/colomemaria/ARID1A_follicular_lymphoma.
