# Supplementary material for: ARID1A mutations protect follicular lymphoma from FAS-dependent immune surveillance by reducing RUNX3/ETS1-driven FAS-expression
Source: Cell Death Differ. 2025 Jan 23;32(5):899–910. doi: 10.1038/s41418-025-01445-3 (PMC12089402; doi:10.1038/s41418-025-01445-3)
Supplement: Supplementary file 1 — Supplementary File [file 41418_2025_1445_MOESM1_ESM.docx]

***ARID1A* Mutations Protect Follicular Lymphoma from FAS-dependent Immune Surveillance by Reducing RUNX3/ETS1-Driven FAS-Expression**

**Running Title:** *ARID1A* Mutations Lower RUNX3/ETS1-Driven FAS Expression in FL

Martina Antoniolli*^1,2^, Maria Solovey*^3^, Johannes Adrian Hildebrand*^1,2,4^, Tabea Freyholdt^1,2^, Carolin Dorothea Strobl^1,2^, Deepak Bararia^1,2^, William David Keay^1,2,4^, Louisa Adolph^1,2^, Michael Heide^1,2,4^, Verena Passerini^1,2^, Lis Winter^2,5^, Lucas Wange^6^, Wolfgang Enard^6^, Susanne Thieme^7^, Helmut Blum^7^, Martina Rudelius^2,8^, Julia Mergner^9^, Christina Ludwig^10^, Sebastian Bultmann^11^, Marc Schmidt-Supprian^4,12^, Heinrich Leonhardt^11^, Marion Subklewe^2,4,5^, Michael von Bergwelt-Baildon^1,2,4,13,14^, Maria Colomé-Tatché*^3,15^, Oliver Weigert*^1,2,4,14^

** These authors contributed equally to this work.*

^1^Laboratory for Experimental Leukemia and Lymphoma Research (ELLF), LMU University Hospital, Munich, Germany.

^2^Department of Medicine III, LMU University Hospital, Munich, Germany.

^3^Biomedical Center (BMC), Department of Physiological Chemistry, Faculty of Medicine, LMU Munich, Planegg-Martinsried, Germany

^4^German Cancer Consortium (DKTK), Munich, Germany; and German Cancer Research Center (DKFZ), Heidelberg, Germany.

^5^Laboratory for Translational Cancer Immunology, Gene Center, LMU Munich, Germany.

^6^Anthropology and Human Genomics, Faculty of Biology, LMU Munich, Planegg, Germany.

^7^Laboratory for Functional Genome Analysis (LAFUGA), Gene Center, LMU Munich, Munich, Germany.

^8^Institute of Pathology, LMU University Hospital, Munich, Germany.

^9^Bavarian Center for Biomolecular Mass Spectrometry at Klinikum Rechts der Isar (BayBioMS@MRI), Technical University Munich, Munich, Germany.

^10^Bavarian Center for Biomolecular Mass Spectrometry (BayBioM), TUM School of Life Science, Technical University Munich, Munich, Germany.

^11^Faculty of Biology and Center for Molecular Biosystems (BioSysM), Human Biology and BioImaging, LMU Munich, Planegg, Germany.

^12^Institute of Experimental Hematology, TranslaTUM, Klinikum rechts der Isar, Technical University Munich, Munich, Germany.

^13^Comprehensive Cancer Center Munich (CCCM), University Hospital, LMU Munich, Germany

^14^Bavarian Cancer Research Centre (BZKF), Munich, Germany.

^15^Institute of Computational Biology, Helmholtz Zentrum Munich, German Research Center for Environmental Health, Neuherberg, Germany.

**Correspondence:** Oliver Weigert, MD, Laboratory for Experimental Leukemia and Lymphoma Research (ELLF), Department of Medicine III, Ludwig-Maximilians-University (LMU) Hospital, Max-Lebsche-Platz 30, D-81377 Munich, Germany. Tel. +49 89 4400 43985. Fax. +49 89 4400 43970, E-Mail [oliver.weigert@med.uni-muenchen.de](mailto:oliver.weigert@med.uni-muenchen.de)

Maria Colomé-Tatché, Ph.D., Department of Physiological Chemistry, Biomedical Center (BMC), Ludwig-Maximilians-University, Grosshaderner Strasse 9, D-82152 Planegg-Martinsried, Munich, Germany. Tel. +49 89 2180 77095, E-Mail maria.colome@bmc.med.lmu.de

**Supplementary Figures**

**
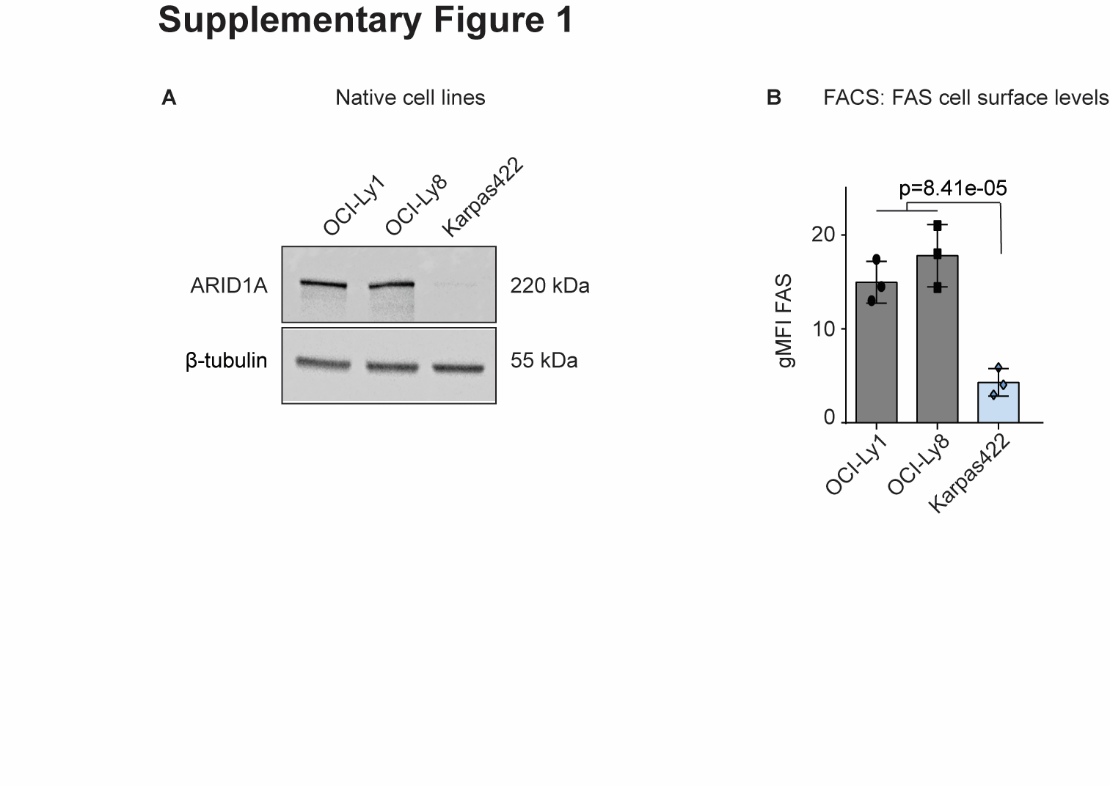
**

**Supplementary Figure 1:** **A** Western blot for ARID1A in *ARID1A*^WT^ OCI-Ly1 and OCI-Ly8 and *ARID1A^MUT^* Karpas422 native cells (N = 1). **B** FAS cell surface expression by FACS on *ARID1A*^WT^ (OCI-Ly1 and OCI-Ly8) and *ARID1A*^MUT^ (Karpas422) native cells. Bar diagram depicting the geometric means of independent replicates (N = 3). *P-value is from two-sided t-test. ARID1A^MUT^ cell lines were tested against ARID1A^WT^ cell lines.* **C** Validation of *FAS* RNA expression by quantitative real-time PCR (TaqMan assay) in OCI-Ly8 and OCI-Ly1 clones (N = 3). *P-values for OCI-Ly1 are from two-sided t-test, for OCI-Ly8 from Mann-Whitney U-test, Bonferroni-adjusted. All groups were tested against WT. Pooled data from biological replicates (N) are represented as mean± SD.*


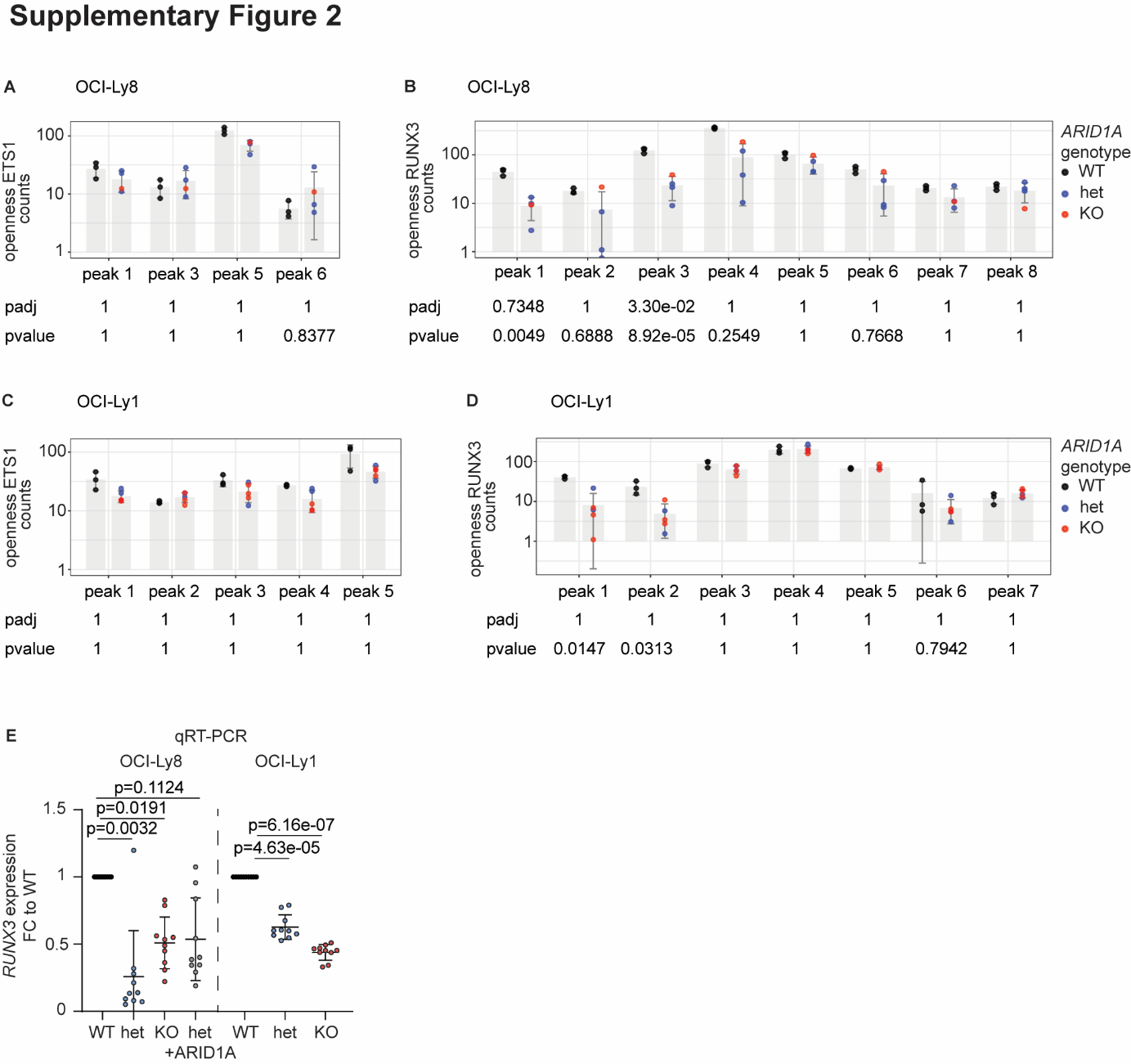


**Supplementary Figure 2:** **A** Chromatin openness of ETS1 in OCI-Ly8. **B** Chromatin openness of RUNX3 in OCI-Ly8. **C** Chromatin openness of ETS1 in OCI-Ly1. **D** Chromatin openness of RUNX3 in OCI-Ly1. Black indicates WT, blue indicates *ARID1A*^het^ and red indicates *ARID1A*^hom^. **E** Validation of *FAS* RNA expression by quantitative real-time PCR in OCI-Ly8 and OCI-Ly1 clones (N = 9). *P-values are from the two-sided t-test, Bonferroni-adjusted. All groups were tested against WT. Pooled data from biological replicates (N) are represented as mean± SD.*


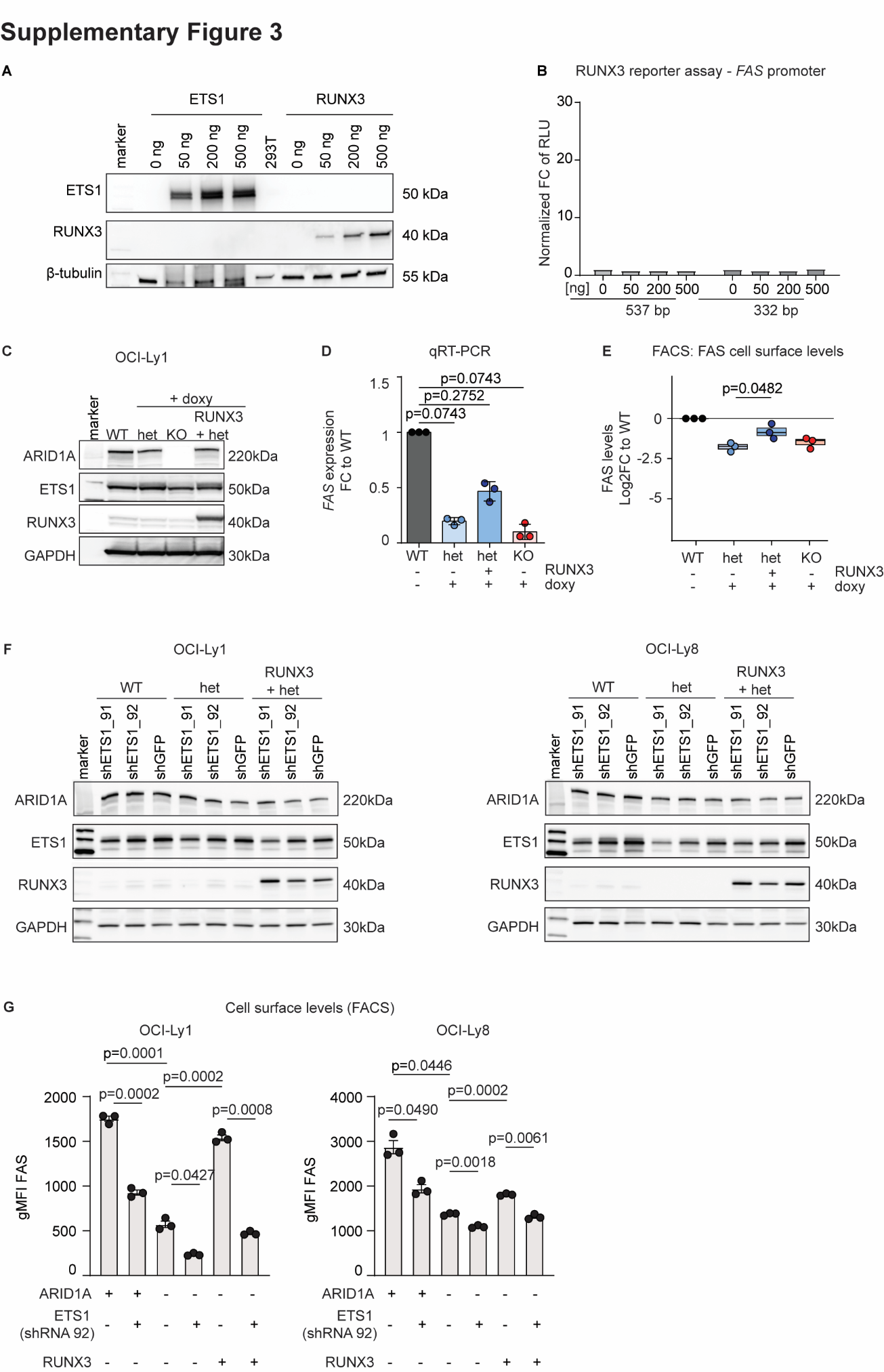


**Supplementary Figure 3:** **A** Western blot of ETS1 and RUNX3 in HEK 293T cells after transfection with increasing doses of the respective expression vectors (N = 1). **B** Luciferase reporter assay, co-transfection of the RUNX3 expression vector and pGL3-FAS constructs (N = 3). **C** Western blot of OCI-Ly1 clones (ARID1A WT, het, and KO) with or without stable doxycycline (dox)-induced overexpression of RUNX3 (N = 3). **D** Rescue of *FAS* RNA levels upon RUNX3 overexpression in OCI-Ly1 measured by quantitative real-time PCR (TaqMan assay) (N = 3). *P-values are from Bonferroni-adjusted Mann-Whitney U-test. All groups were tested against WT.* **E** Rescue of FAS cell-surface protein levels upon RUNX3 overexpression in OCI-Ly1 measured by FACS (N = 3). *P-value is from two-sided t-test. Het+RUNX3 was tested against het. Pooled data from biological replicates (N) are represented as mean*± *SD.* **F** Western blot for ARID1A, ETS1 and RUNX3 in OCI-Ly1 and OCI-Ly8 cells after stable expression of ETS1-targeting shRNAs (shETS1_91 and shETS1_92) and doxycycline-induced RUNX3 overexpression (N = 2). **G** FAS surface expression comparing ARID1AWT (“+”), ARID1Ahet (“-“) with and without ETS1-targeting shRNA (shRNA92) with and without RUNX3 overexpression in OCI-Ly1 (left bar plot) and OCI-Ly8 (right bar plot) cells by FACS (N = 3). *P-values are from paired Welch-test, Bonferroni adjusted.*

**Graphical abstract**

Low FAS expression in *ARID1A* mutant lymphoma cells is mediated by reducing RUNX3/ETS1-driven FAS transcription and promotes a functionally and potentially clinically relevant escape from T cell mediated killing.

**Supplementary Tables**

**Table S1.** List of differentially accessible peaks on promoters.

**Table S2.** List of labeled peaks on *FAS*, *RUNX3*, and *ETS1*.

**Table S3.** List of transcription factors involved in *FAS* regulation.

**Table S4.** List of co-transcription factors involved in *FAS* regulation.

**Table S5.** List of oligonucleotides.

**Table S6.** List of antibodies.

**Table S7.** List of differentially expressed genes.

**Table S8.** List of indexes used for ATAC-sequencing.
