# Supplementary material for: ARID1A mutations protect follicular lymphoma from FAS-dependent immune surveillance by reducing RUNX3/ETS1-driven FAS-expression
Source: Cell Death Differ. 2025 Jan 23;32(5):899–910. doi: 10.1038/s41418-025-01445-3 (PMC12089402; doi:10.1038/s41418-025-01445-3)

Figure 2A

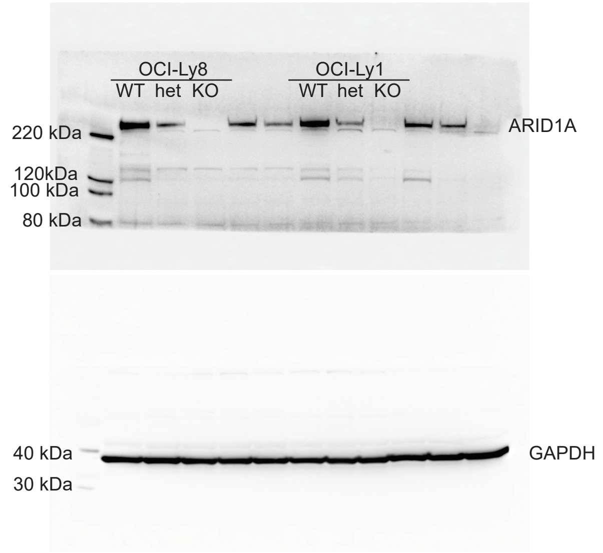

Figure 4D

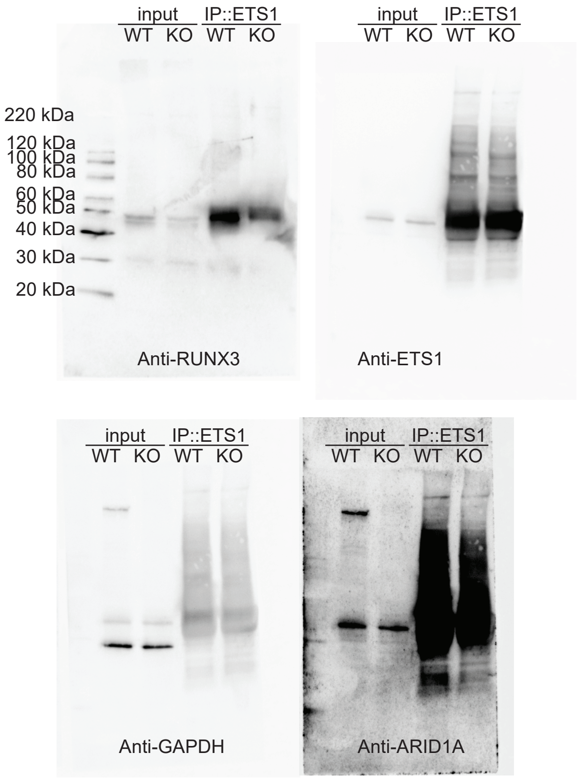

Figure 4E

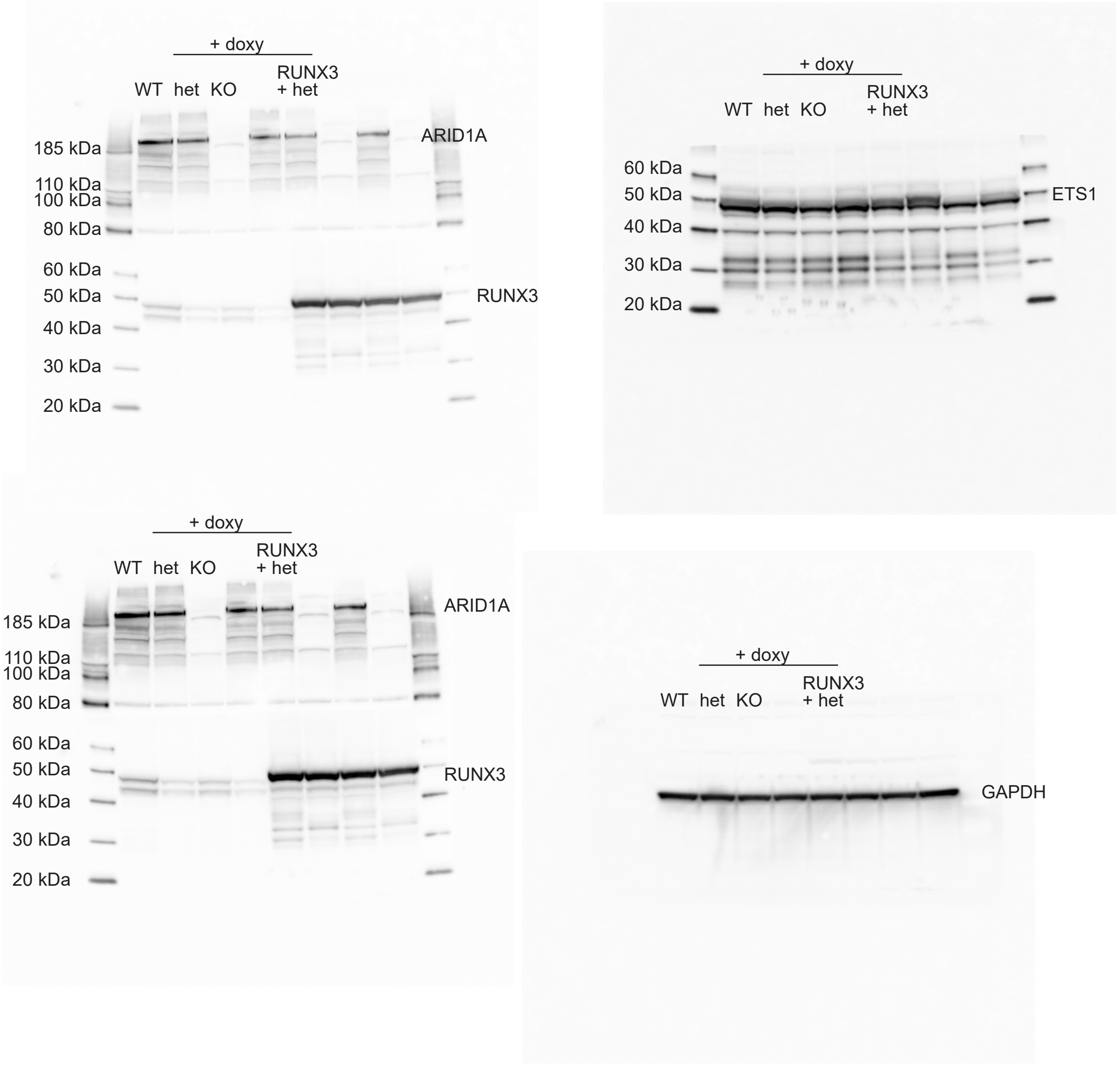

Supplementary Figure 1A

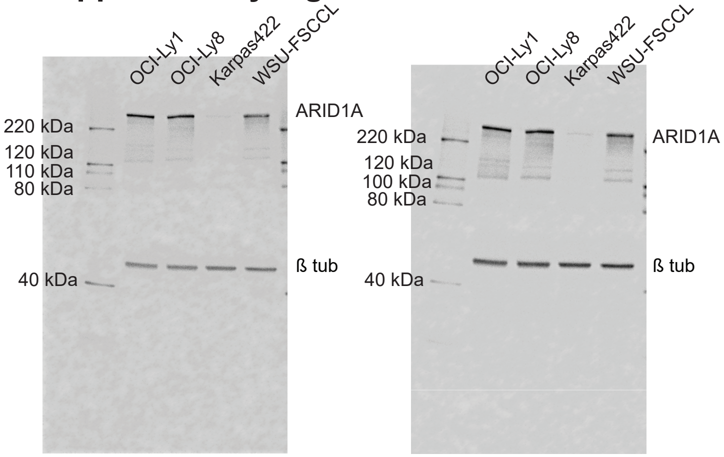

Supplementary Figure 3A

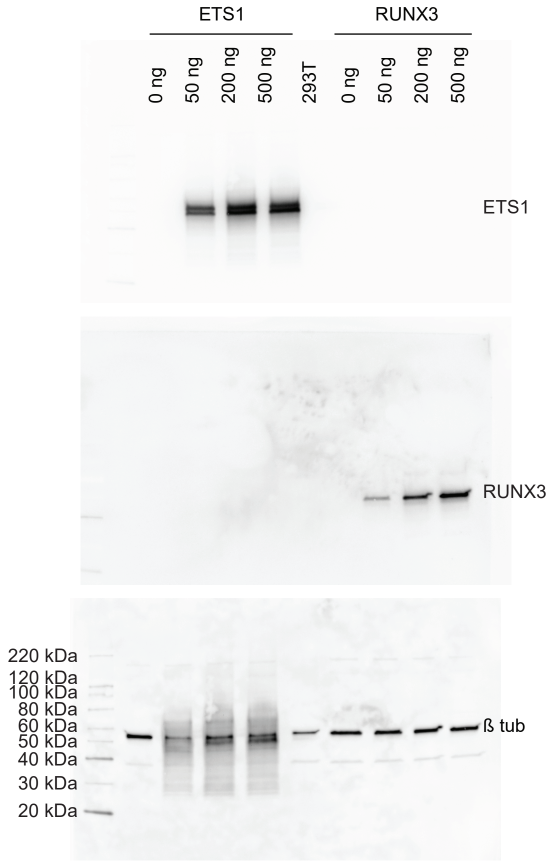

Supplementary Figure 3C

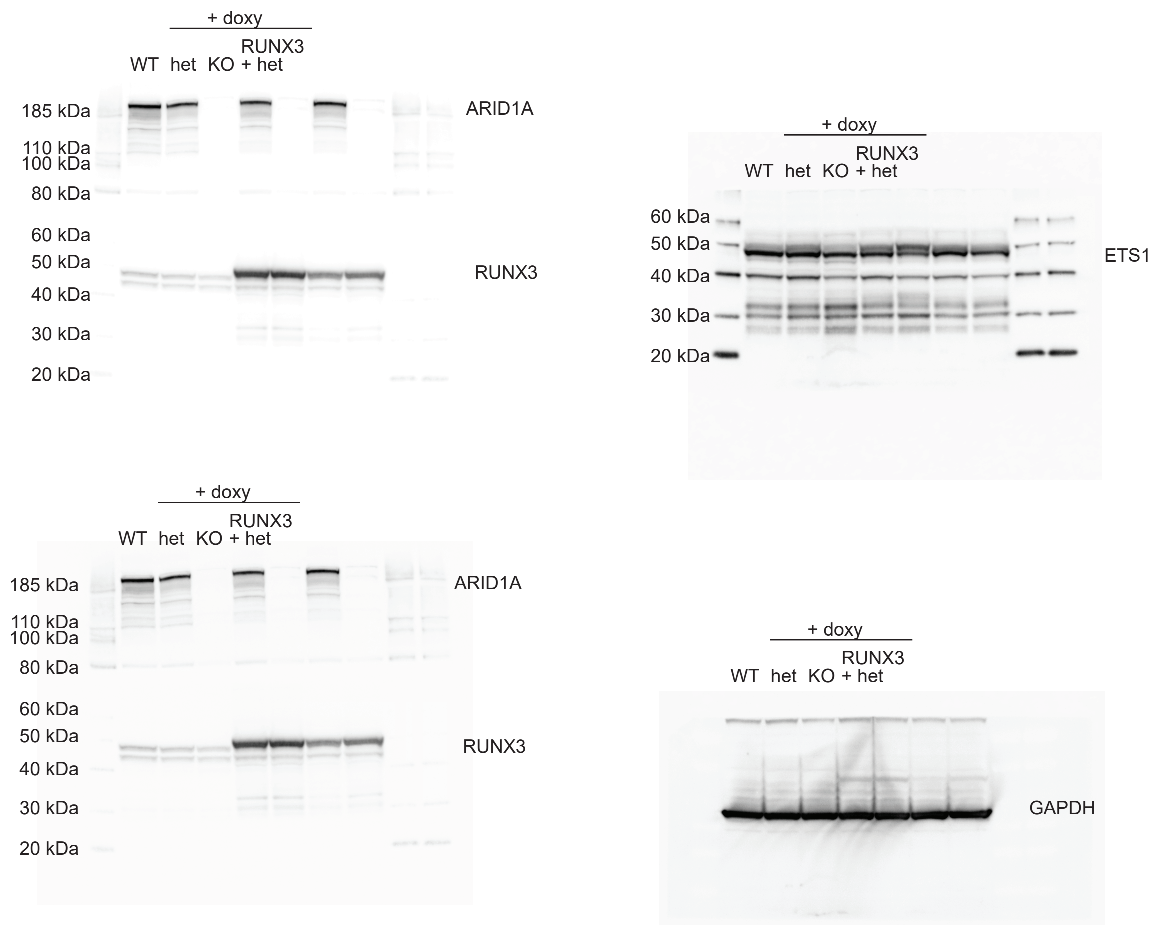

Supplementary Figure 3F

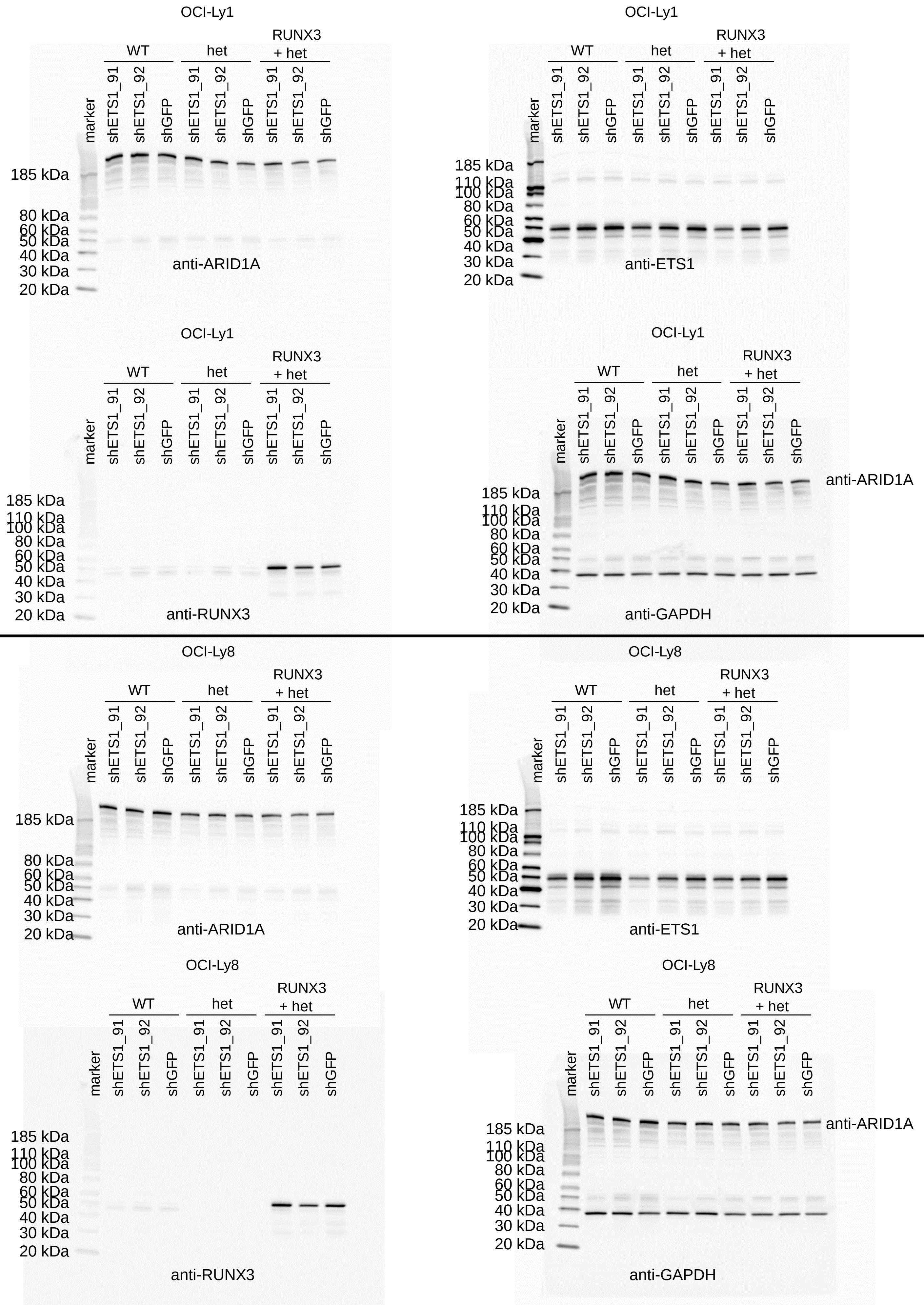

Supplement: Supplementary file 10 — Supplemental Figure WB full lenght [file 41418_2025_1445_MOESM10_ESM.pdf]
